# *Azospirillum brasilense* improves rice growth under salt stress by regulating the expression of key genes involved in salt stress response, abscisic acid signaling, and nutrient transport, among others

**DOI:** 10.3389/fagro.2023.1216503

**Published:** 2023-10-04

**Authors:** Zachariah Degon, Seth Dixon, Yasir Rahmatallah, Mary Galloway, Sophia Gulutzo, Hunter Price, John Cook, Galina Glazko, Arijit Mukherjee

**Affiliations:** 1 Department of Biology, University of Central Arkansas, Conway, AR, United States,; 2 Department of Biomedical Informatics, University of Arkansas for Medical Sciences, Little Rock, AR, United States

**Keywords:** rice, salt stress, plant growth-promoting bacteria, *Azospirillum brasilense*, RNA-seq

## Abstract

Major food crops, such as rice and maize, display severe yield losses (30–50%) under salt stress. Furthermore, problems associated with soil salinity are anticipated to worsen due to climate change. Therefore, it is necessary to implement sustainable agricultural strategies, such as exploiting beneficial plant-microbe associations, for increased crop yields. Plants can develop associations with beneficial microbes, including arbuscular mycorrhiza and plant growth-promoting bacteria (PGPB). PGPB improve plant growth via multiple mechanisms, including protection against biotic and abiotic stresses. *Azospirillum brasilense*, one of the most studied PGPB, can mitigate salt stress in different crops. However, little is known about the molecular mechanisms by which *A. brasilense* mitigates salt stress. This study shows that total and root plant mass is improved in *A. brasilense*-inoculated rice plants compared to the uninoculated plants grown under high salt concentrations (100 mM and 200 mM NaCl). We observed this growth improvement at seven- and fourteen days post-treatment (dpt). Next, we used transcriptomic approaches and identified differentially expressed genes (DEGs) in rice roots when exposed to three treatments: 1) *A. brasilense*, 2) salt (200 mM NaCl), and 3) *A. brasilense* and salt (200 mM NaCl), at seven dpt. We identified 786 DEGs in the *A. brasilense*-treated plants, 4061 DEGs in the salt-stressed plants, and 1387 DEGs in the salt-stressed *A. brasilense*-treated plants. In the *A. brasilense*-treated plants, we identified DEGs involved in defense, hormone, and nutrient transport, among others. In the salt-stressed plants, we identified DEGs involved in abscisic acid and jasmonic acid signaling, antioxidant enzymes, sodium and potassium transport, and calcium signaling, among others. In the salt-stressed *A. brasilense*-treated plants, we identified some genes involved in salt stress response and tolerance (e.g., abscisic acid and jasmonic acid signaling, antioxidant enzymes, calcium signaling), and sodium and potassium transport differentially expressed, among others. We also identified some *A. brasilense*-specific plant DEGs, such as nitrate transporters and defense genes. Furthermore, our results suggest genes involved in auxin and ethylene signaling are likely to play an important role during these interactions. Overall, our transcriptomic data indicate that *A. brasilense* improves rice growth under salt stress by regulating the expression of key genes involved in defense and stress response, abscisic acid and jasmonic acid signaling, and ion and nutrient transport, among others. Our findings will provide essential insights into salt stress mitigation in rice by *A. brasilense*.

## 1 Introduction

Abiotic factors such as salt, heat, and drought stresses, and nutrient deficiency are responsible for extensive crop loss and soil degradation, resulting in an estimated $27B annual loss (Pitman and Lauchli, 2002; Hoang et al., 2016; Zorb et al., 2019). For example, salt stress is a primary abiotic stress, estimated to impact over 20% of irrigated agricultural land, with >50% of arable land anticipated to be salt affected by 2050 (Wang et al., 2003; Munns and Tester, 2008; Jamil et al., 2011). The abundance of soil salinization stems from multiple elements, including inadequate agricultural practices (e.g., irrigation), land degradation (e.g., evaporates), and adverse climatic conditions (e.g., drought, rising sea levels, etc.). Furthermore, the accumulation of salt in the soil leads to salt and drought stress responses in plants that drastically impede the plant’s fitness, with effects ranging from decreased yield, decreased nutrient acquisition, and impeded root and shoot development to cell oxidation, nutrient imbalance, ion toxicity, and chlorophyll degradation (Munns and Tester, 2008; Shahbaz and Ashraf, 2013). Therefore, it is imperative to implement sustainable agricultural strategies to limit the loss in global crop production due to these stresses.

Rice is one of the most susceptible crops to high salt concentrations (Munns and Tester, 2008; Hanin et al., 2016; Hoang et al., 2016). It is estimated that rice yield is reduced by almost 30–50% annually due to salt stress, and climate change will likely worsen it (Eynard et al., 2004). So naturally, numerous studies have been conducted on rice to investigate the physiological responses and molecular mechanisms under high salt concentrations (100–250 mM NaCl) (Li et al., 2017; Chandran et al., 2019; Liu et al., 2019). These studies in rice have identified several salt stress-responsive and -tolerance genes, contributing to our understanding of the genetic pathways(s) regulating the plant’s response and adaptation to salt stress (Kawasaki et al., 2001; Chandran et al., 2019; Razzaque et al., 2019; Mansuri et al., 2020; Farooq et al., 2021). However, developing salt-tolerant crops through transgenic technologies and conventional breeding approaches can be labor-intensive and time-consuming. Furthermore, some salt-tolerant plants developed using these approaches have had limited success under field saline conditions (Jamil et al., 2011). Therefore, one option is to simultaneously utilize alternative approaches to promote sustainable agriculture, such as using plant-beneficial microbes.

Plants can benefit from associations with multiple microbes, including mycorrhizal fungi and plant growth-promoting bacteria (PGPB) (Santi et al., 2013; Pankievicz et al., 2019). Several studies have shown that PGPB can promote plant growth via biological nitrogen fixation, hormone synthesis, protection against biotic and abiotic stresses, phosphate solubilization, iron sequestration, etc. (Glick, 2012; Olanrewaju et al., 2017; Backer et al., 2018; Pankievicz et al., 2021). As a result, some of these PGPB (e.g., *Azospirillum*, *Burkholderia*, *Herbaspirillum*) are already used as an inoculum in agriculture worldwide. In addition, studies in different plant systems (e.g., wheat, rice, *Arabidopsis thaliana*) have reported that inoculation with PGPB such as *A. brasilense* and *P. phytofirmans* can promote growth and nutrient uptake under high salinity conditions (Hamdia et al., 2004; Pinedo et al., 2015; Ilangumaran and Smith, 2017; Kumar et al., 2020; Kruasuwan et al., 2023). However, little is known about how these PGPB mediate salt stress in the host plants at a molecular level.

Previously, we established an experimental system in which *A. brasilense* Sp245 can promote rice growth under *in-vitro* conditions (Thomas et al., 2019). In the current study, using this system, we investigated if *A. brasilense* Sp245 could promote rice (Nipponbare cv.) growth under high salt concentrations (100 mM and 200 mM NaCl). We hypothesized that *A. brasilense* would improve rice growth under high salt stress and regulate host gene expression. We also hypothesized that the expression pattern of rice genes involved in stress and defense responses, hormone signaling, and nutrient transport would be regulated during this process. Understanding the underlying molecular mechanisms via which *A. brasilense* improves rice growth under salt stress will be vital as we develop strategies to grow crops under harsh environmental conditions.

## 2 Materials and methods

### 2.1 Plant material, growth conditions, salt treatment, and bacterial inoculation

The plant preparation and growth conditions in this study were similar to previous studies (Hiltenbrand et al., 2016; Thomas et al., 2019; Wiggins et al., 2022). First, the wild-type rice (*Oryza sativa* cv. Nipponbare) seeds were surface-sterilized in a 70% (v/v) sodium hypochlorite solution for approximately fifteen minutes, rinsed five times with distilled water and then imbibed overnight (no less than 12hr) in sterile, distilled water. Next, the sterilized rice seeds were placed onto sterile germination paper (Anchor Paper, Saint Paul, MN, USA) placed in 9-cm Petri plates (#633185, Greiner bio-one, Monroe, NC, USA), sealed with parafilm (L-2020-1, BioExpress, Kaysville, UT, USA), and allowed to germinate in the dark for approximately 72–96 hrs. The germinated seedlings were transferred onto 15-cm Petri plates (#639102, Greiner bio-one, Monroe, NC, USA) containing low-N_2_ Fahraeus medium (FM) and grown inside a Percival growth chamber (#CU-22L, Perry, IA, USA) with a 16-h, 22°C day and 8-h, 24°C night cycle and 150–200 μmol m^−2^s^−1^ light intensity, with 65% humidity for seven days. Next, the rice seedlings were transferred to new sterile 15-cm Petri dishes containing FM supplemented with different amounts of NaCl (0-, 100-, and 200 mM). These salt concentrations represent high salt stress and have been used in previous salt stress studies in rice (Kawasaki et al., 2001; Li et al., 2017; Chandran et al., 2019; Liu et al., 2019). The rice roots were inoculated with or without *A. brasilense* Sp245 as done previously (Thomas et al., 2019; Wiggins et al., 2022). Briefly, bacteria were grown on Tryptone Yeast Extract (TY) media at 30° C to an optical density (600 nm) of 0.6 (Schnabel et al., 2010; Bible et al., 2015; Mitra et al., 2016). Next, the bacterial cells were resuspended in sterile water before the rice seedlings were inoculated with 10^8^ cells/ml of *A. brasilense* Sp245. We used sterile water as a mock treatment (control) in these experiments. The plants were grown in the Percival growth chamber under the abovementioned conditions for seven or fourteen days before the plant mass phenotypes were recorded. These experiments included at least three biological replicates. Plant masses were individually analyzed via One-Way ANOVA using JMP v15 software (SAS Institute Inc., NC, USA) among all four treatments: control, salt only, *A. brasilense* only, *A. brasilense* + salt. For *post-hoc* analysis, planned comparisons were designed as follows: control treatment vs. salt-only treatment, control treatment vs. *A. brasilense*-only treatment, and Salt-only treatment vs. *A. brasilense* + salt treatments.

### 2.2 RNA-sequencing and data analysis

Briefly, the samples for the RNA-seq experiment include: (a) rice (Nipponbare cv.) + mock inoculation; (b) rice (Nipponbare cv.) + *Azospirillum brasilense*; (c) rice (Nipponbare cv.) + 200 mM NaCl treatment; (d) rice (Nipponbare cv.) + *Azospirillum brasilense +* 200 mM NaCl treatment. Total RNA was extracted from rice roots seven days post-treatment (dpt) from the abovementioned treatment groups using Qiagen RNeasy^®^ Plant Mini Kit (Cat #74904, California, USA) per manufacturer’s protocol. The RNA samples were then treated with Ambion^®^ DNA-free^™^ DNase Treatment and Removal (Cat #AM1906, California, USA) kit, and RNA concentrations and purities were measured using the NanoDrop 2000 spectrophotometer (Thermo Scientific, Delaware, USA). Three biological replicate samples were obtained for each treatment group.

Total RNA isolated from the different samples were sent to Novogene Genomics Services and Solutions (CA, USA) for the RNA integrity tests, library preparation, and sequencing. The messenger RNA was purified from total RNA samples using poly-T oligo-attached magnetic beads for poly-A enrichment. The libraries were checked with Qubit and real-time PCR for quantification and bioanalyzer for size distribution detection. The quantified libraries were pooled and sequenced on Illumina Novaseq platform, and paired-end reads were generated. The raw sequence data is publicly available from the Sequence Read Archive (SRA) under the BioProject accession ID PRJNA962515. Data analysis was performed as done previously (Thomas et al., 2019; Wiggins et al., 2022). Briefly, raw 150 base pairs paired-end reads were processed using *Trimmomatic* (version 0.39) (Bolger et al., 2014) to remove Illumina adapter and PCR primer sequences, remove leading and trailing bases with a low-quality score, trim read ends if the average quality per base drops below a specified threshold (Phred score 15), and drop reads shorter than 50 base pairs after applying all filtering steps. Reads were aligned to the rice genome (*Oryza sativa*) using *Tophat* (version 2.0.12) (Trapnell et al., 2009) allowing two base pairs mismatches per read (default parameter). Aligned reads were quantified by gene loci and normalized to fragments per kilobase of transcripts per million mapped reads (FPKM) values using cufflinks (version 2.2.1) (Trapnell et al., 2010). *Oryza sativa* reference genome (version 7) and gene annotations for 55986 loci were obtained from the Rice Genome Annotation Project (Kawahara et al., 2013). Differential expression analysis was performed using *cuffdiff* (part of the Cufflinks software), and significant differentially expressed genes (DEGs) were defined as those with false discovery rate (FDR) <0.05 and absolute fold-change greater than 1.5 (|FC|>1.5). We performed three comparisons: 1) rice + mock inoculation (or control) against rice + *A. brasilense* inoculation; 2) rice + mock inoculation (or control) against rice + 200 mM NaCl treatment; 3) rice + mock inoculation (or control) against rice + 200 mM NaCl treatment + *A. brasilense* inoculation. The DEGs in each of the three comparisons were further tested for over-represented gene ontology (GO) terms to associate differential expression profiles with biological interpretations.

### 2.3 Reverse-transcriptase- PCR

We validated the expression of eight genes identified via RNA-seq using reverse-transcriptase polymerase chain reaction (RT-PCR). Genes validated via RT-PCR include brassinosteroid insensitive-1 receptor-like kinase, phytosulfokine precursor, WRKY27 transcription factor, MYB-family transcription factor, ent-Kaurene synthetase, naringenin, 2-oxoglutarate 3-deoxygenase, bZIP transcription factor, and cam1-calmodulin kinase. Three cDNA sets of each treatment group were synthesized from 300 ng of pure RNA samples using Thermo Scientific RevertAid RT Kit (#K1691) with Oligo(dT)_18_ primers per manufacturer’s instructions. Before cDNA synthesis, we processed the RNA samples with Ambion^®^ DNA-free DNase Treatment and Removal kit (Cat #AM1906, Foster City, CA, USA). The primers for the RT-PCR are included in Supplementary Table 5. We used *Cyclophilin* as an internal reference gene in these experiments. These experiments were performed in at least three biological replicates.

## 3 Results

### 3.1 *Azospirillum brasilense* inoculation improves rice growth when grown under high salt concentrations

**FIGURE 1:**
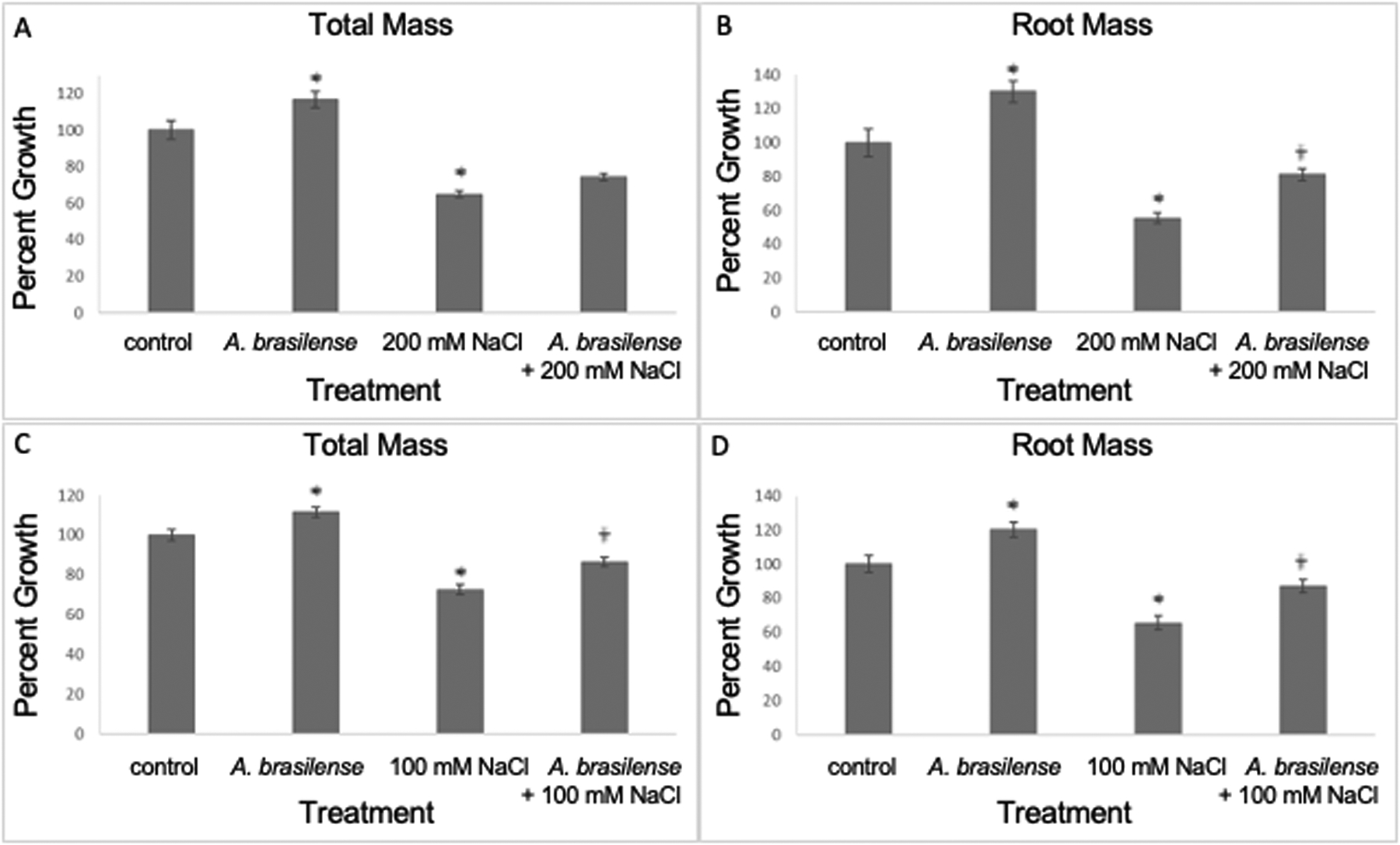
Inoculation with *A*. *brasilense* improves rice growth under high salt concentrations, seven days post treatment. There were significant increases in plant mass (total and root mass) when treated with *A*. *brasilense* in contrast to the control treatment [one-way ANOVA, F_3,265_ = 45.0 **(A)** and 30.3 **(B)**, p<0.0001; planned contrast, p=0.0019 **(A)** and p=0.0006 **(B)**; one-way ANOVA, F_3,245_ = 35.0 **(C)** and 23.9 **(D)**, p<0.0001; planned contrast, p=0.0012 **(C)** and p=0.0009 **(D)**]. There were significant decreases in plant mass when subjected to high-salt treatments (100 and 200 mM NaCl) in contrast to the control treatment [one-way ANOVA, F_3,265_ = 45.0 **(A)** and 30.3 **(B)**, p<0.0001; planned contrast, p<0.0001 **(A, B)**; one-way ANOVA, F_3,245_ = 35.0 **(C)** and 23.9 **(D)**, p<0.0001; planned contrast, p<0.0001 **(C, D)**]. There were significant increases in *A*. *brasilense*-treated root mass subjected to 200 mM NaCl in contrast to plants solely subjected to the 200 mM NaCl treatment [one-way ANOVA, F_3,265_ = 45.0 **(A)** and 30.3 **(B)**, p<0.0001; planned contrast, p=0.0567 **(A)** and p=0.0014 **(B)**]. There were significant increases in *A*. *brasilense*-treated plant mass subjected to 100 mM NaCl in contrast to plants solely subjected to the 100 mM NaCl treatment [one-way ANOVA, F_3,245_ = 35.0 **(C)** and 23.9 **(D)**, p<0.0001; planned contrast, p=0.002 **(C)** and p=0.0034 **(D)**]. Asterisks (*) denote p<0.05 in contrast to the control treatment; Obelisk denotes p<0.05 in contrast to the high-salt treatment (mean ± SE, n>45 per treatment and obtained in at least three experimental replicates).

We investigated if *A. brasilense* Sp245 could improve growth in rice plants when grown under high-salt concentrations (100 mM and 200 mM NaCl). At seven days post-treatment (dpt), our results show that the total and root plant mass increased in *A. brasilense*-treated salt-stressed (200 mM NaCl) plants when compared to salt-stressed (200 mM NaCl) plants (one-way ANOVA, F_3,265_ = 45.0 and 30.3, p<0.0001; planned contrast, p=0.0567 and p=0.0014, respectively) (Figures 1A, B). In addition, at seven dpt, the total and root plant mass increased in *A. brasilense*-treated salt-stressed (100 mM NaCl) plants when compared to salt-stressed (100 mM NaCl) plants (one-way ANOVA, F_3,245_ = 35.6 and 23.9, p<0.0001; planned contrast, p=0.002 and p=0.0034, respectively) (Figures 1C, D). Other treatments included plants exposed to salt stress (200 mM and 100 mM NaCl) only, plants inoculated with *A. brasilense* but not exposed to salt stress, and plants exposed to mock treatment (water) (Figure 1). As expected, plants exposed to salt stress (200 mM and 100 mM NaCl) only had significant reduction in total and root mass compared to the mock controls (one-way ANOVA, F_3,265_ = 45.0 and 30.3, p<0.0001; planned contrast, p<0.0001) (Figures 1A, B); (one-way ANOVA, F_3,245_ = 35.6 and 23.9, p<0.0001; planned contrast, p<0.0001, respectively) (Figures 1C, D). Similarly, plants treated with *A. brasilense* had significant increase in mass compared to the mock controls (one-way ANOVA, F_3,265_ = 45.0 and 30.3, p<0.0001; planned contrast, p=0.0019 and p=0.0006, respectively) (Figures 1A, B); (one-way ANOVA, F_3,245_ = 35.6 and 23.9, p<0.0001; planned contrast, p=0.0011 and p=0.0006, respectively) (Figures 1C, D).

**FIGURE 2:**
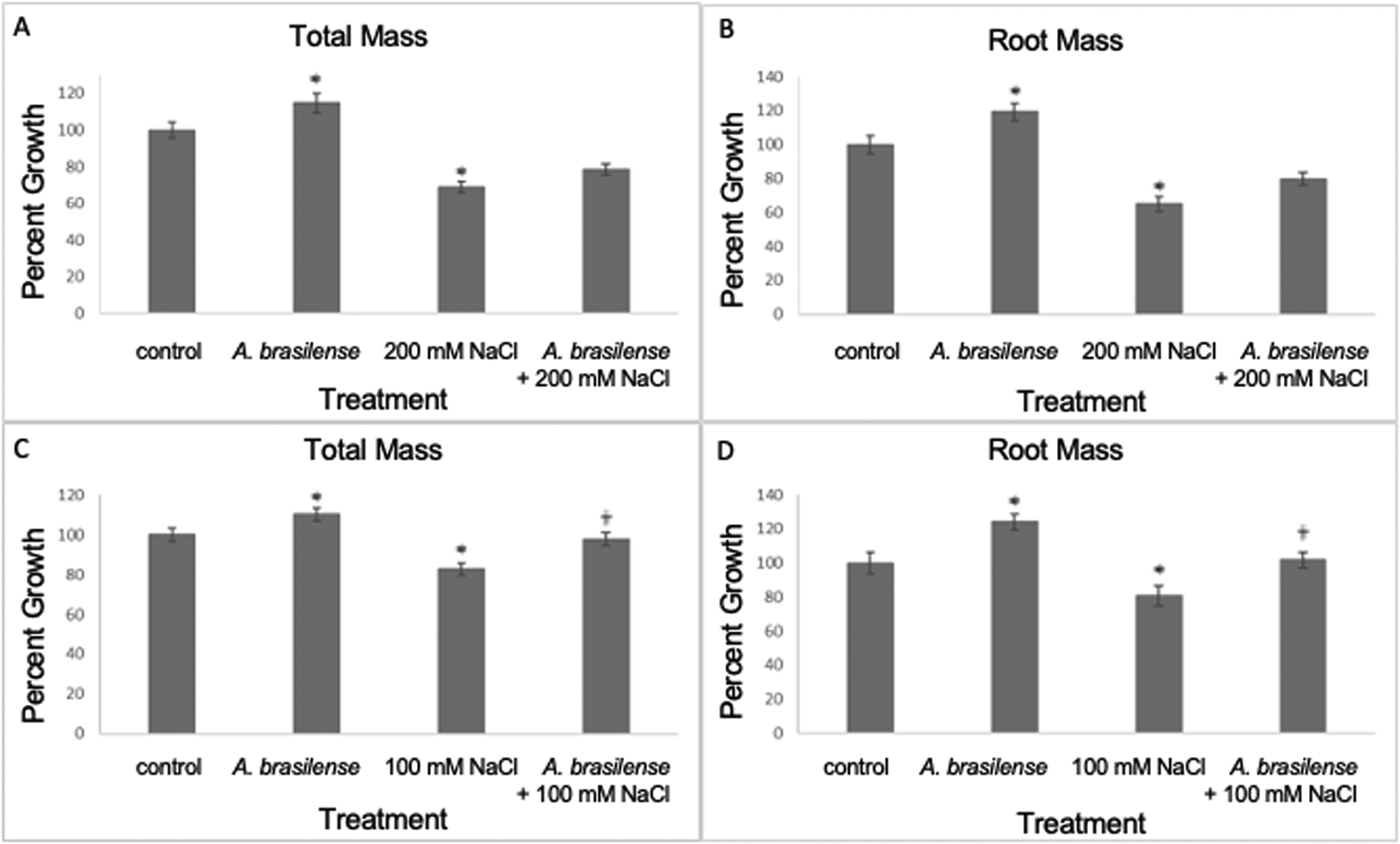
Inoculation with *A. brasilense* improves rice growth under high salt concentrations, fourteen days post treatment. There were significant increases in plant mass (total and root mass) when treated with *A. brasilense* in contrast to the control treatment [one-way ANOVA, F_3,258_ = 24.8 **(A)** and 13.9 **(B)**, p<0.0001; planned contrast, p=0.01 **(A, B)**; one-way ANOVA, F_3,169_ = 13.96 **(C)** and 11.94 **(D)**, p<0.0001; planned contrast, p=0.0247 **(C)** and p=0.019 **(D)**]. There were significant decreases in plant mass subjected to both high-salt treatments in contrast to the control treatment (one-way ANOVA, F_3,258_ = 24.8 **(A)** and 13.9 **(B)**, p<0.0001; planned contrast, p=0.00045 **(A)** and p=0.0001 **(B)**; one-way ANOVA, F_3,169_ = 13.96 **(C)** and 11.94 **(D)**, p<0.0001; planned contrast, p=0.0001 **(C)** and p=0.0091 **(D)**). There was an increase in mass (total and root mass) of rice plants that were *A. brasilense*-treated and subjected to 200 mM NaCl in contrast to plants solely subjected to the 200 mM NaCl treatment [one-way ANOVA, F_3,258_ = 24.8 **(A)** and 13.9 **(B)**, p<0.0001; planned contrast, p=0.087 **(A)** and p=0.1 **(B)**]. There was a significant increase in mass (total and root mass) of rice plants that were *A. brasilense*-treated and subjected to 100 mM NaCl in contrast to plants solely subjected to the 100 mM NaCl treatment [one-way ANOVA, F_3,169_ = 13.96 **(C)** and 11.94 **(D)**, p<0.0001; planned contrast, p=0.0007 **(C)** and p=0.0039 **(D)**]. Asterisks (*) denote p<0.05 in contrast to the control treatment; Obelisk denotes p<0.05 in contrast to the high-salt treatment (mean ± SE, n>50 per treatment and obtained in at least three experimental replicates).

Next, we investigated if *A. brasilense* could improve plant growth at a later time point, fourteen dpt, in rice plants when grown under high-salt concentrations (100 mM and 200 mM NaCl). At fourteen (dpt), our results show that the total and root plant mass increased in *A. brasilense*-treated salt-stressed (200 mM NaCl) plants when compared to salt-stressed (200 mM NaCl) plants (one-way ANOVA, F_3,258_ = 24.8 and 13.9, p<0.0001; planned contrast, p=0.087 and p=0.1, respectively) (Figures 2A, B). In addition, at fourteen dpt, the total and root plant mass increased in *A. brasilense*-treated salt-stressed (100 mM NaCl) plants when compared to salt-stressed (100 mM NaCl) plants (one-way ANOVA, F_3,169_ = 13.9 and 11.9, p<0.0001; planned contrast, p=0.0007 and p=0.0039, respectively) (Figures 2C, 1D). Other treatments included plants exposed to salt stress (200 mM and 100 mM NaCl) only, plants inoculated with *A. brasilense* but not exposed to salt stress, and plants exposed to mock treatment (water) (Figure 2). Like earlier, the plants exposed to salt stress (200 and 100 mM NaCl) had a significant reduction in total and root mass compared to the mock controls (one-way ANOVA, F_3,258_ = 24.8 and 13.9, p<0.0001; planned contrast, p<0.0001 and p=0.0001, respectively) (Figures 2A, B); (one-way ANOVA, F_3,169_ = 13.9 and 11.9, p<0.0001; planned contrast, p=0.0001 and p=0.0091, respectively) (Figures 2C, D). Similarly, plants treated with *A. brasilense* had a significant increase in mass compared to the mock controls (one-way ANOVA, F_3,258_ = 24.8 and 13.9, p<0.0001; planned contrast, p=0.0099 and p=0.01, respectively) (Figures 2A, B); (one-way ANOVA, F_3,169_ = 13.9 and 11.9, p<0.0001; planned contrast, p=0.0247 and p=0.0019, respectively) (Figures 2C, D). In conclusion, our results show that *A. brasilense* inoculation improved growth in rice plants under salt stress.

### 3.2 The rice root transcriptomes under the different treatments

**FIGURE 3:**
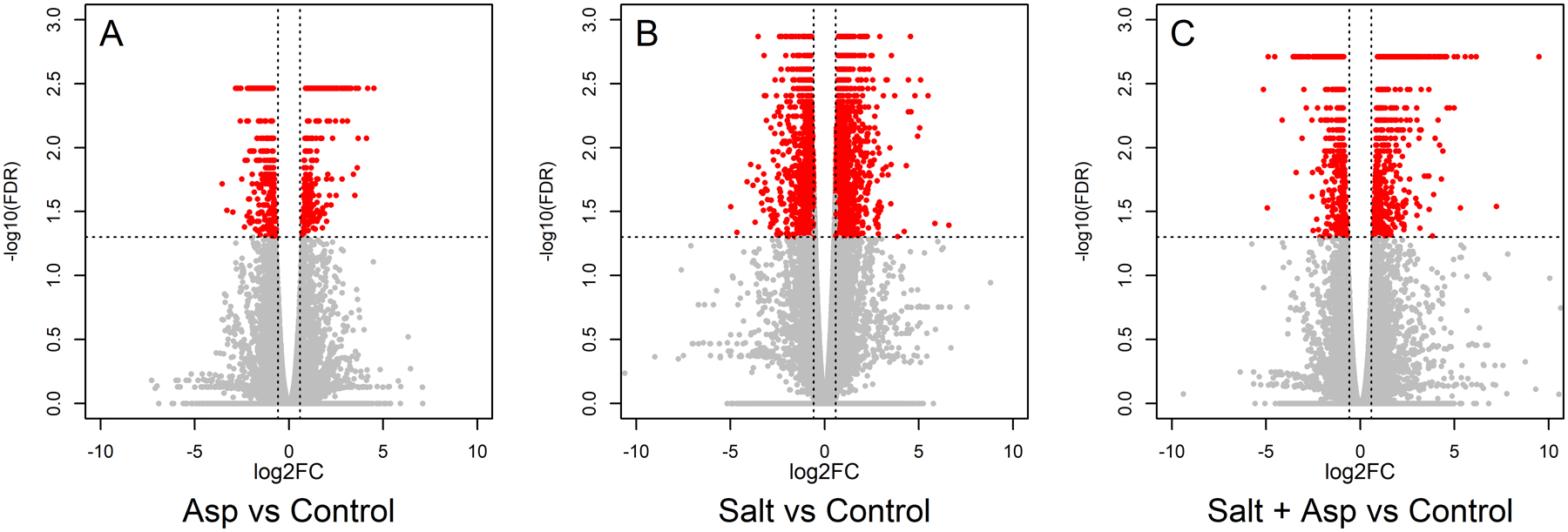
Volcano plots representing the differential expression analysis where mean log_2_ fold change is plotted against the -log_10_(FDR) adjusted P-values for all the expressed genes: **(A)** *A*. *brasilense*-treated versus control samples, **(B)** Salt-stressed versus control samples, and **(C)** *A*. *brasilense*-treated salt-stressed versus control samples. Each dot represents one gene where significant DEGs (FDR adjusted P-value < 0.05 and |FC|>1.5) are represented in red and non-differentially expressed genes are represented in gray color.

Utilizing RNA sequencing, we identified differentially expressed genes (DEGs) in rice roots for these samples, (a) rice (Nipponbare cv.) + *Azospirillum brasilense* vs. rice (Nipponbare cv.) + mock inoculation (water only); (b) rice (Nipponbare cv.) + 200 mM NaCl vs. rice (Nipponbare cv.) + mock inoculation (water only); (c) rice (Nipponbare cv.) + *Azospirillum brasilense +* 200 mM NaCl vs. rice (Nipponbare cv.) + mock inoculation (water only). We collected the data at seven dpt, and each sample included three biological replicates. Sequencing was performed using a 150 base pairs paired-end approach on an Illumina NovaSeq platform. An average of 22 million raw reads were obtained per sample, of which 96% survived quality control. The filtered reads had an 89.6% average mapping rate to the rice genome (Michigan State University, version 7) (Table 1). We identified the DEGs in the different comparisons using the thresholds FDR-adjusted P-value of <0.05 and absolute fold change of 1.5. For the *A. brasilens*e-treated samples we identified 786 DEGs (Figure 3A and Supplementary Table 1), for the salt-stressed samples we identified 4061 DEGs (Figure 3B and Supplementary Table 2), and for the *A. brasilense*-treated salt-stressed samples we identified 1387 DEGs (Figure 3C and Supplementary Table 3).

**FIGURE 4:**
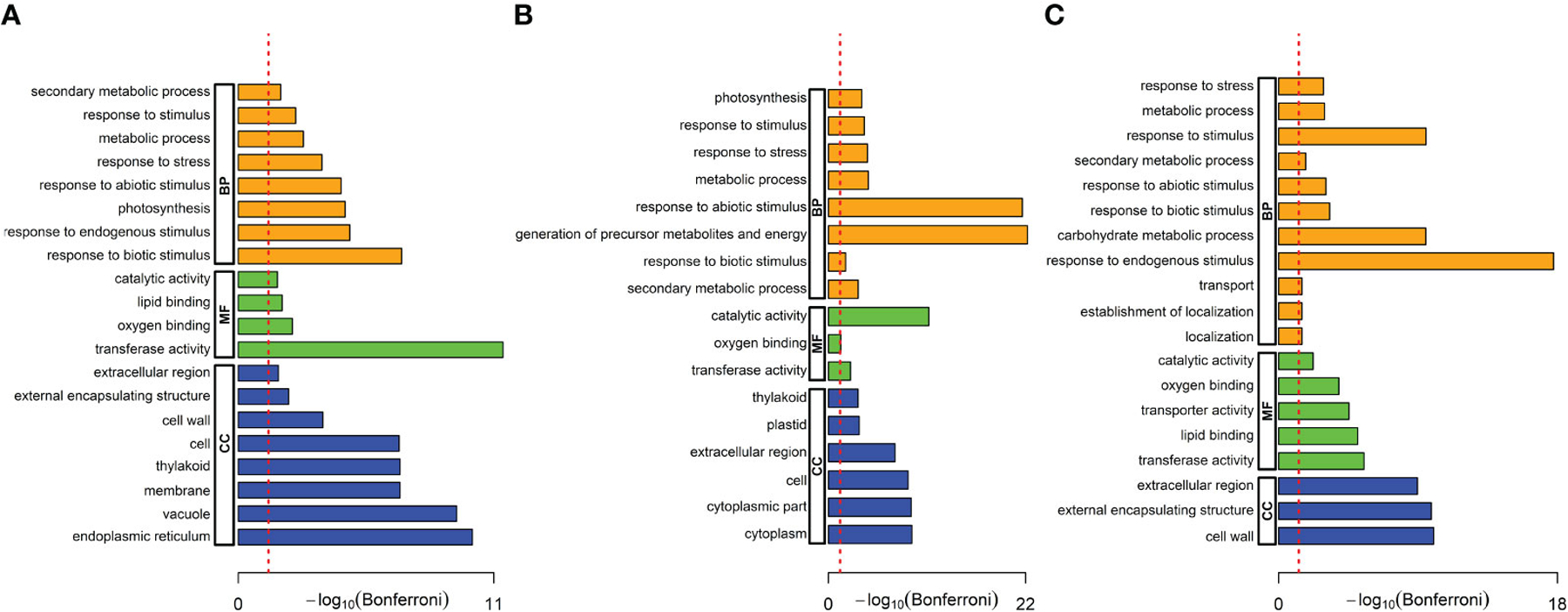
Gene ontology (GO) terms over-represented in the significant DEGs. **(A)** *A*. *brasilense*-treated versus control samples, **(B)** Salt-stressed versus control samples, **(C)** *A*. *brasilense*-treated salt-stressed versus control samples. The X-axis indicates the -log_10_ (FDR) adjusted P-value and the dotted red line indicates the threshold FDR=0.05. Biological processes (BP), molecular functions (MF), and cellular components (CC) terms are respectively represented by orange, green, and blue bars.

We performed a gene ontology (GO) analysis to understand the biological significance of these DEGs. Using AgriGO, we analyzed the DEGs for over-represented biological processes (BP), molecular functions (MF), and cellular components (CC) terms (Tian et al., 2017). First, we identified twenty GO terms that were significantly enriched in *A. brasilense*-treated rice only. These included eight enriched in biological processes (e.g., response to biotic stimulus, response to endogenous stimulus, photosynthesis, response to stimulus, metabolic process, secondary metabolic process, etc.), four in molecular functions (e.g., catalytic activity, lipid binding, oxygen binding, and transferase activity), and eight in cellular components (e.g., endoplasmic reticulum, cell, cell wall, extracellular region, external encapsulating structure, etc.) (Figure 4A). Next, we identified seventeen GO terms that were significantly enriched in rice under the salt-stress treatment only. These included eight enriched in biological processes (e.g., response to abiotic stress, generation of precursor metabolites and energy, secondary metabolic process, photosynthesis, etc.), three in molecular functions (e.g., catalytic activity, transferase activity, and oxygen binding), and six in cellular components (e.g., cytoplasm, extracellular region, cell, thylakoid, etc.) (Figure 4B). Finally, we identified nineteen GO terms that were significantly enriched in *A. brasilense*-treated salt-stressed rice. These included eleven enriched in biological processes (e.g., carbohydrate metabolic process, response to endogenous response, response to stimulus, secondary metabolic process, metabolic process, etc.), five in molecular functions (e.g., transferase activity, lipid binding, transporter activity, oxygen binding, and catalytic activity), and three in cellular components (e.g., extracellular region, cell wall, and external encapsulating structure) (Figure 4C).

We identified 786 DEGs in rice roots during associations with *A. brasilense*, seven dpt (Supplementary Table 1). Among these were several defense-related genes (e.g., pathogenesis-related genes, defensins, thionins, chitinases, and cinnamoyl-CoA-reductase) and genes involved in the flavonoid pathway (e.g., naringenin synthase, chalcone synthase, chalcone-flavonone isomerase, and flavanol synthase). We also identified the differential expression of transcription factors (TFs) (e.g., MYB, WRKY, AP2/ERFs), protein kinases (PK) (e.g., calcium/calmodulin-dependent kinases, SHR5 receptor-like kinases, and wall-associated kinases), and transporters (e.g., nodulins, sugar transporters, amino acid and peptide transporters, ammonium transporters, and high-affinity nitrate transporters). Finally, we identified some hormone-related genes involved in auxin, gibberellin, ethylene, and phytosulfokine signaling in this dataset.

We identified 4061 DEGs in rice roots when grown under salt stress (200 mM NaCl), seven dpt (Supplementary Table 2). Among them were several defense-related genes (e.g., chitinase genes, thionin genes, and defensin genes) and hormonal genes involved in stress responses and regulation (e.g., abscisic acid (ABA), jasmonic acid (JA)). We also identified the differential expression of transcription factors (TFs) (e.g., AP2/ERFs, BHLH, WRKY, MYB, bZIP, and zinc-finger families). Finally, we identified differential expression of antioxidant genes (e.g., catalase, peroxidase, and superoxide dismutase (SOD)) and key ion-transporters (e.g., high-affinity sodium transporters (HKT1/2), cation transporters, and potassium transporter proteins).

We identified 1387 DEGs in rice roots exposed to salt stress (200 mM NaCl) and treated with *A. brasilense*, seven dpt (Supplementary Table 3). Among them was the differential expression of genes in the flavonoid biosynthesis pathway (e.g., naringenin synthase, chalcone synthase, and flavonone synthase), several hormone-related genes (e.g., GH3, phytosulfokines, auxin efflux carriers, auxin-responsive genes, ethylene insensitive 2 (EIN2), 1-aminocyclopropane-1carboxylate (ACC) oxidase, and cytokinin-O-glucosyltransferases), transcription factors (TFs) (e.g., WRKY, bZIP, MYB, zinc fingers), and protein kinases (PK) (e.g., calcium/calmodulin-dependent kinases, wall-associated kinases, and SHR5 receptor-like kinases). In addition, we identified several antioxidant genes (e.g., APX, catalases, and peroxidases) and transporters (e.g., sugar transporters, high-affinity nitrate transporters, ammonium transporters, nodulins, auxin efflux carriers, and ion transporters).

### 3.3 Comparison of the rice transcriptomes under different treatments

Next, we compared the DEGs in the different comparisons to identify the underlying gene expression trends. We performed the following comparisons: 1) DEGs identified in salt-stressed rice vs. DEGs identified in *A. brasilense*-treated salt-stressed rice, and 2) DEGs identified in *A. brasilense*-treated rice vs. DEGs identified in *A. brasilense*-treated salt-stressed rice.

**FIGURE 5:**
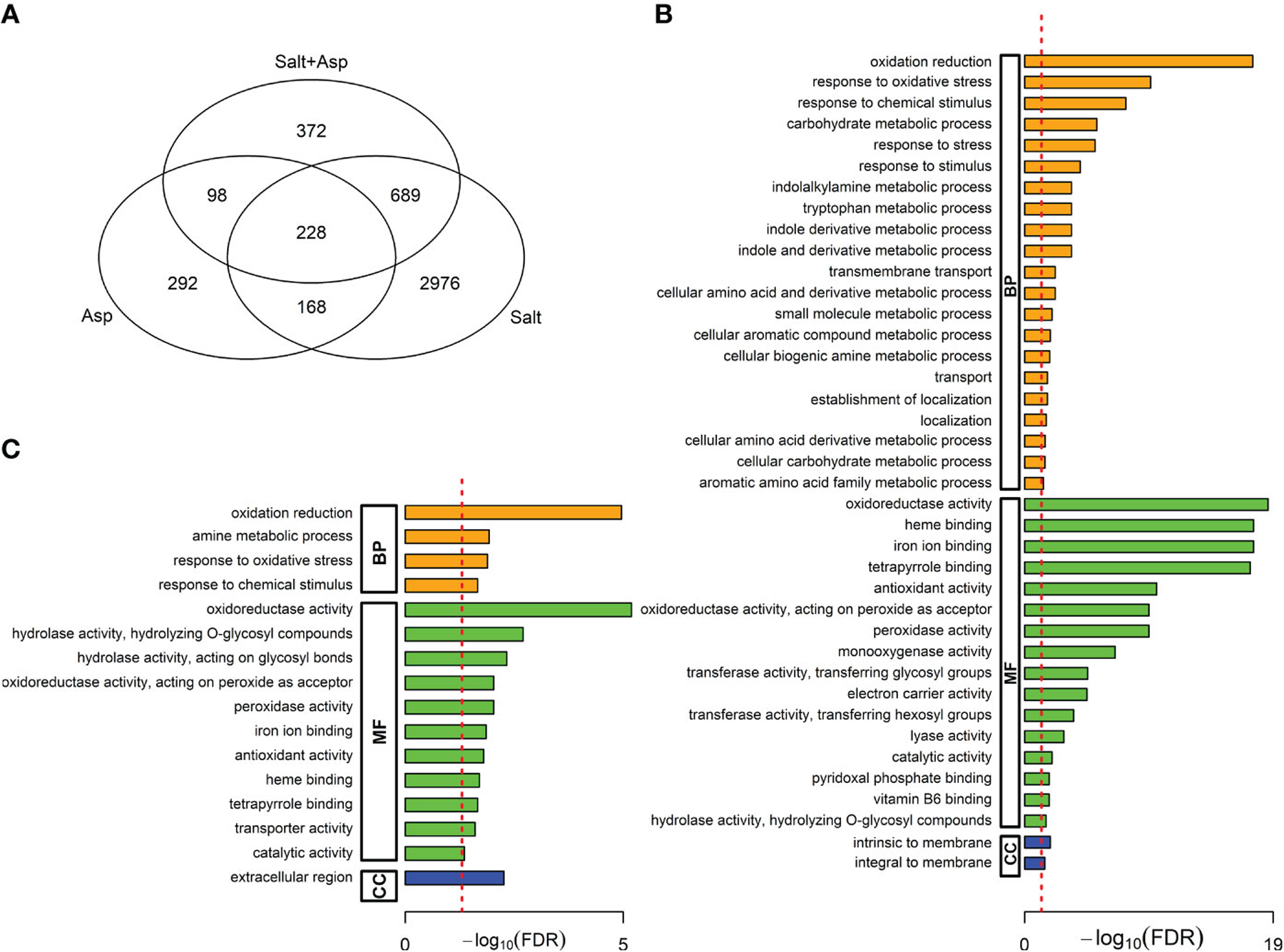
**(A)** Venn diagram showing the intersections among the significant differentially expressed genes (DEGs) in the three comparisons: *A*. *brasilense*-treated versus controls, salt-stressed versus controls, and *A*. *brasilense*-treated salt-stressed versus controls, **(B)** Gene Ontology (GO) terms over-represented in 689 DEGs common in salt-stressed versus controls and *A*. *brasilense*-treated salt-stressed versus controls, but not in *A*. *brasilense*-treated versus controls, **(C)** Gene Ontology (GO) terms over-represented in 98 DEGs common in *A*. *brasilense*-treated versus controls and *A*. *brasilense*-treated salt-stressed versus controls, but not in salt-stressed versus controls.

In the first comparison, we identified 917 genes differentially expressed in both treatments (Figure 5A). Among these, 228 genes were differentially expressed under all three treatments, and 689 genes were differentially expressed in salt-stressed plants, and *A. brasilense*-treated salt-stressed plants (Supplementary Table 4). The list of 689 DEGs included numerous salt stress-response and tolerance genes, including genes involved in abscisic acid and jasmonic acid signaling, genes encoding antioxidant enzymes, and genes involved in sodium and potassium transport and calcium signaling, among others (Supplementary Table 4). In addition, we performed gene ontology (GO) analysis and identified GO terms involved in response to oxidative stress, response to stress, and transmembrane transport, among others (Figure 5B).

In the second comparison, we identified 326 genes differentially expressed in both treatments (Figure 5A). Among these, 228 genes were differentially expressed under all three treatments, and 98 genes were differentially expressed in plants treated with only *A. brasilense*, and *A. brasilense*-treated salt-stressed plants (Supplementary Table 4). The list of 98 DEGs included genes involved in defense and stress response, hormone signaling pathways, flavonoid biosynthesis pathway, and nutrient transporters such as nitrate, ammonium, and sugar transporters, among others (Supplementary Table 4). In addition, we performed gene ontology (GO) analysis and identified GO terms involved in oxidoreductase activity, antioxidant activity, and transporter activity, among others (Figure 5C).

### 3.4 Validation of gene expression patterns via RT-PCR

**FIGURE 6:**
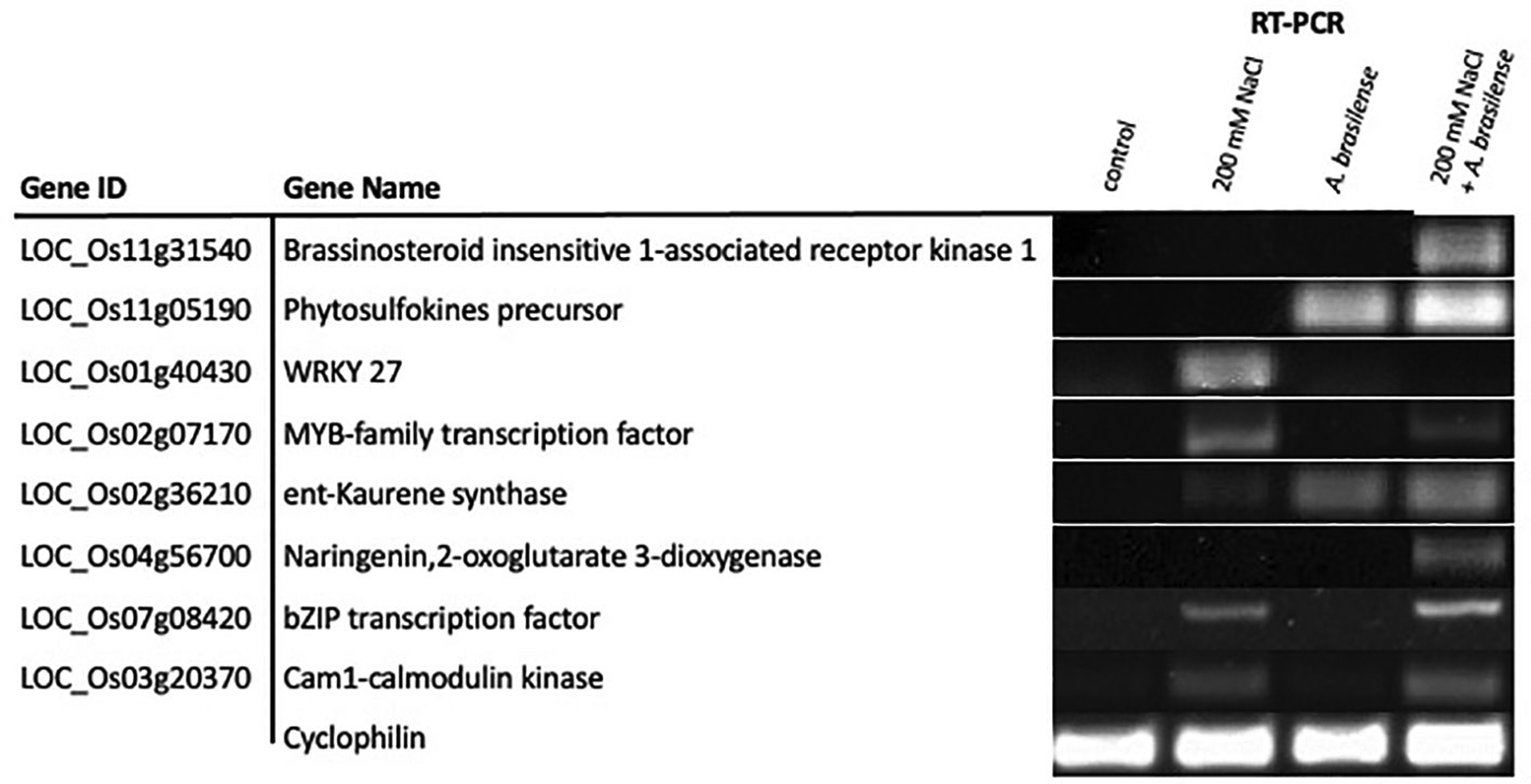
RT-PCR validation of differentially expressed genes identified via RNA-Seq. Expression patterns of eight DEGs were validated via RT-PCR. For the RT-PCR experiments, control, 200 mM NaCl, *A. brasilense*, and 200 mM NaCl + *A. brasilense* represent the cDNA templates synthesized from their respective RNA samples. *Cyclophilin* was used as internal reference gene. RT-PCR was performed in at least three biological replicates for all samples.

Lastly, we validated the expression patterns of eight differentially expressed genes among our three treatment groups. Genes validated via RT-PCR include brassinosteroid insensitive-1 receptor-like kinase, phytosulfokine precursor, WRKY27 transcription factor, MYB-family transcription factor, ent-Kaurene synthetase, naringenin 2-oxoglutarate 3-deoxygenase, bZIP transcription factor and cam1-calmodulin kinase (Figure 6). Our RT-PCR results confirm the gene expression trends observed in the RNA-seq experiment.

## 4 Discussion

Soil salinity is considered one of the most limiting factors for agricultural productivity and food security (FAO, 2015; FAO et al., 2022). In fact, rice is one of the most susceptible crops to high salt concentrations and experiences a significant yield reduction due to salinity stress (Eynard et al., 2004; Munns and Tester, 2008; Rapaport et al., 2013; Hanin et al., 2016; Hoang et al., 2016). Several studies have shown that PGPB can improve salt tolerance in plants and promote their growth (Hamdia et al., 2004; Pinedo et al., 2015; Ilangumaran and Smith, 2017; Kumar et al., 2020; Kruasuwan et al., 2023). However, the underlying molecular mechanisms by which PGPB improve salt stress tolerance are largely unknown. In this study, we show that the PGPB *Azospirillum brasilense* improves rice growth under high salt-stress conditions. Furthermore, we used a transcriptomic approach to identify the genetic pathways contributing to *A. brasilense*-mediated salt tolerance in rice. Below we discuss some of our findings from this study.

Before proceeding with the gene expression experiments, we investigated if *A. brasilense* could improve salt tolerance in rice under our experimental conditions. So first, we showed that the high-salt (100 mM and 200 mM NaCl) treatments impeded plant growth in rice, seven and fourteen dpt. These results were expected, as we utilized a salt-susceptible rice cultivar, Nipponbare cv., that has been shown to display severe yield losses under moderate-high salt concentrations (Karan et al., 2012; Shankar et al., 2016). Next, we showed that *A. brasilense* inoculation improved rice growth at seven and fourteen dpt, as shown previously (Thomas et al., 2019). Finally, we showed that rice plant mass improved in *A. brasilense*-treated salt-stressed plants compared to salt-stressed (100 and 200 mM NaCl) uninoculated plants at seven and fourteen dpt. Unsurprisingly, our results showed a greater improvement in plant mass in *A. brasilense*-treated salt-stressed plants compared to salt-stressed uninoculated plants with the lower salt concentration (100 mM NaCl) at both time points. However, our results also indicate that at seven dpt, the salt treatment has a more drastic effect on plant mass than at fourteen dpt at both concentrations. This was expected as several glycophytic crops are most susceptible to salt stress at early developmental stages (Horie et al., 2001; Munns and Tester, 2008). The same applies to *A. brasilense*-treated salt-stressed plants, so over time the detrimental effect of salt is reduced under these conditions. These results suggest that *A. brasilense* inoculation played a role in improving salt stress tolerance in rice. However, the plant growth promotion effects observed in *A. brasilense*-treated salt-stressed plants were still lower than in *A. brasilense*-treated plants not exposed to salt stress. Nevertheless, we established an experimental system to investigate the molecular basis of plant growth promotion by *A. brasilense* when rice is grown under salt-stress conditions.

Next, we performed an RNA-seq experiment to identify the regulation of gene expression in rice roots upon three different treatments: 1) *A. brasilense* only, 2) 200 mM NaCl only, and 3) *A. brasilense* + 200 mM NaCl. For this study, we collected the transcriptomic data at seven dpt, as we observed that *A. brasilense* could improve rice growth under high salt concentration at this time point. We selected the higher concentration, 200 mM, of NaCl treatment as we observed that *A. brasilense* could mitigate salt stress in rice even at this concentration. In this study, we focused on rice roots as these tissues are the first point of interaction between the host plant and the symbiotic bacteria. Future studies can focus on identifying the transcriptomic responses in plant shoots to obtain a holistic understanding of the transcriptomic changes in the host plant. We identified hundreds of DEGs under each treatment. In this study, we focused on genes previously implicated in salt response and tolerance in rice and rice- *A. brasilense* interaction and compared their expression profiles across the treatments. Below we discuss some of these findings.

### 4.1 Genes involved in salt stress response and tolerance

Several reports have described the transcriptional responses in rice roots upon salt stress exposure (Kumar et al., 2013; Shankar et al., 2016; Zhou et al., 2016; Chandran et al., 2019; Ma et al., 2022). These studies identified stress-responsive genes, transcription factors, genes involved in hormone signaling, and sodium and potassium transport differentially expressed in salt-stressed rice plants. In this study, we identified several salt stress response genes differentially expressed in rice roots treated with 200 mM NaCl. We also examined how their expression was regulated in rice roots upon *A. brasilense* treatment (*A. brasilense* only and *A. brasilense* + salt) (Supplementary Table 6).

#### 4.1.1 Genes involved in ABA and JA signaling

Studies have shown that osmotic stress due to high salt concentration increases abscisic acid (ABA) biosynthesis, subsequently regulating gene expression in the ABA signaling pathway (Kumar et al., 2013; Sah et al., 2016; Huang et al., 2021). In this study, we identified two phytoene synthase genes (LOC_Os12g43130 and LOC_Os06g51290), a 9-cis-epoxycarotenoid dioxygenase gene (LOC_Os07g05940), and a zeaxanthin epoxidase gene (LOC_Os04g37619) upregulated in salt-stressed plants. The 9-cis-epoxycarotenoid dioxygenase gene (LOC_Os07g05940) displayed higher expression compared to the other genes. These genes have been shown to be expressed in rice upon exposure to salt stress, and their expression correlated to the level of ABA in rice roots (Li et al., 2008; Welsch et al., 2008; Farooq et al., 2021). None of these genes were expressed in rice roots treated with only *A. brasilense*. However, it was interesting to observe none of these genes were expressed in *A. brasilense*-treated salt-stressed rice. The expression pattern suggests that genes involved in the ABA signaling pathway in response to salt stress are altered upon *A. brasilense* treatment. Besides the ABA signaling pathway, salt stress response activates the jasmonate signaling pathway in several plants, including *Arabidopsis* and rice (Riemann et al., 2015; Valenzuela et al., 2016; Delgado et al., 2021). For instance, salt stress causes increased levels of jasmonic acid in plant tissues and induces the expression of genes involved in jasmonic acid signaling (Delgado et al., 2021). In this study, we identified two genes encoding for jasmonate-induced proteins (LOC_Os04g24328 and LOC_Os04g24319) upregulated in expression in rice roots exposed to salt stress. However, these genes were not expressed in salt-stressed plants treated with *A. brasilense*, suggesting the PGPB treatment alters the regulation of genes from the jasmonate signaling pathway that mediate salt stress responses.

#### 4.1.2 Genes encoding antioxidant enzymes

Salt stress in plants leads to oxidative stress, causing reactive oxygen species (ROS) accumulation and reduced plant growth (Qu et al., 2010; Hasanuzzaman et al., 2021). Plants deal with the adverse effects of oxidative stress via antioxidant enzymes such as catalases, glutathione-S-transferases (GST), and copper-zinc superoxide dismutases (SOD) (Choudhury et al., 2017). Several studies in different plants have shown differential expression of genes encoding these antioxidant enzymes during salt stress (Baltruschat et al., 2008; Vighi et al., 2017). In this study, we observed the upregulation in the expression of genes encoding catalases (LOC_Os03g03910 and LOC_Os02g02400), GSTs (e.g., LOC_Os01g49710, LOC_Os10g34020, LOC_Os01g49720, LOC_Os06g08670), and SODs (LOC_Os07g46990, LOC_Os08g44770, LOC_Os03g11960) in salt-stressed plants. However, the majority of these genes were not expressed in *A. brasilense*-treated salt-stressed plants. A similar expression pattern of genes encoding antioxidant enzymes was also detected in salt-susceptible rice cultivar IR29 upon inoculation with PGPB *Streptomyces* sp. GKU 895 (Kruasuwan et al., 2023). The expression pattern of these genes suggests the PGPB treatment relieves stress in the host plant exposed to high salt concentrations.

#### 4.1.3 Genes involved in sodium and potassium transport, and calcium signaling

Several genes involved in sodium and potassium ion transport are differentially expressed in salt-stressed plants (Kumar et al., 2013; Zhou et al., 2016; Assaha et al., 2017; Zhang et al., 2018). The sodium transporter genes from the HKT family have been shown to play a role in salt tolerance in rice (Horie et al., 2001; Ren et al., 2005; Horie et al., 2007; Zhou et al., 2016). We identified *HKT1* (LOC_Os01g20160) and *HKT2* (LOC_Os06g48810) genes downregulated in rice exposed to salt treatment only. The rice potassium channels, *AKT1* and *SKOR*, have also been suggested to be involved in salt tolerance (Golldack et al., 2003; Fuchs et al., 2005; Musavizadeh et al., 2021). Previously, it was reported *AKT1* expression decreased in rice roots exposed to 150 mM NaCl (Fuchs et al., 2005). We identified the *AKT1* (LOC_Os01g45990) and *SKOR* (LOC_Os04g36740) potassium channels downregulated in salt-treated rice roots. Interestingly, *HKT1*, *HKT2*, and *AKT1* were not differentially expressed in *A. brasilense*-treated plants, but *SKOR* was upregulated in *A. brasilense*-treated plants. In *A. brasilense*-treated salt-stressed plants, *HKT2* was upregulated in expression, but *HKT1* or *SKOR* were not differentially expressed. Taken together, the transcriptomic data suggest that *A. brasilense* inoculation regulates the expression of sodium and potassium transporters in salt-stressed plants. Studies have revealed that calcium signaling affects plant responses to salt stress (Kader and Lindberg, 2010; Manishankar et al., 2018). Accumulating evidence suggests the involvement of a diverse array of calcium sensor proteins in different aspect of salt tolerance (Kim et al., 2007; Seifikalhor et al., 2019). Calcineurin is a calcium and calmodulin-dependent serine/threonine phosphatase mediating salt stress tolerance in rice (Ma et al., 2005). In this study, we identified two calcineurin genes (LOC_Os02g18930 and LOC_Os01g41510) downregulated in rice roots exposed to salt stress only. However, these genes were not differentially expressed in *A. brasilense*-treated salt-stressed rice roots. We also identified a calmodulin-related calcium sensor protein (LOC_Os04g41540) upregulated in expression in salt-stressed rice roots, but not expressed in *A. brasilense*-treated salt-stressed plants. The sodium/calcium exchanger protein family plays an important role in cellular calcium homeostasis (Singh et al., 2015). While their functions are not completely understood in plants, emerging evidence suggests these play a role during stress responses. For instance, in rice, some of these genes were induced in expression by salt stress (Singh et al., 2015; Yang et al., 2021). We identified two genes encoding sodium/calcium exchanger proteins (LOC_Os01g37690 and LOC_Os02g21009) differentially expressed in salt-stressed rice, but these were not expressed in rice treated with *A. brasilense*. These results suggest *A. brasilense* inoculation also modulates the expression pattern of genes involved in calcium sensing and homeostasis during salt stress.

#### 4.1.4 Other salt -responsive and -tolerance genes

Recently using a genome-wide meta-analysis, including microarray and RNA-seq data, one study identified several promising candidate genes for salt tolerance in rice (Mansuri et al., 2020). This list included a CBS domain-containing membrane protein (LOC_Os2g06410) and an expansin precursor gene (LOC_Os05g39990) involved in plant cell wall organization (Mansuri et al., 2020). We noticed these genes were expressed in salt-stressed plants, with the CBS domain-containing membrane protein (LOC_Os2g06410) having a higher expression than the expansin precursor gene (LOC_Os05g39990). However, these genes were not expressed in plants treated with *A. brasilense*. Pentatricopeptide repeat (PPR) proteins are one of the largest protein families in plants and have been reported to be involved in plants’ response to different abiotic stresses, including salt (Li et al., 2021). One recent study showed that the PPR-domain protein SOAR1 regulates salt tolerance in rice (Lu et al., 2022). We identified several genes encoding PPR proteins (e.g., LOC_Os06g31300, LOC_Os10g10170, LOC_Os03g02430, LOC_Os03g11020) upregulated in salt-stressed plants. Interestingly, these genes were not expressed in *A. brasilense*-treated salt-stressed plants. The dehydration-responsive element binding (*DREB*) proteins are key regulators of abiotic stresses in plants (Zhao et al., 2016; Song et al., 2021). One recent study showed *DREB* genes promote tolerance to heat, drought, and salt in rice (Wang et al., 2022). We identified several *DREB* genes (e.g., LOC_Os09g35010, LOC_Os09g35030) upregulated in expression in salt-stressed rice. However, these genes were not differentially expressed in *A. brasilense*-treated salt-stressed rice.

Transcription factors (TF) families such as MYBs, WRKYs, ARFs, and zinc fingers have been shown to be involved in mediating salt stress in plants (Kumar et al., 2013; Shankar et al., 2016; Zhou et al., 2016; Chandran et al., 2019). Some of these TFs were also differentially expressed in rice IR29 under salt stress and *Streptomyces* sp. GKU 895 treatments (Kruasuwan et al., 2023). In this study, we observed the expression of MYBs (e.g., LOC_Os12g39640, LOC_Os07g02800, LOC_Os01g09280), WRKYs (e.g., LOC_Os06g30860, LOC_Os05g46020, LOC_Os01g60600), and zinc fingers (e.g., LOC_Os05g10670, LOC_Os06g15330, LOC_Os08g03310) in rice plants exposed to salt stress. Another study reported that some of these TFs (e.g., LOC_Os07g02800, LOC_Os01g60600, LOC_Os05g10670) were expressed in rice (Japonica cultivar Chilbo) roots treated with 250 mM NaCl for five days (Chandran et al., 2019). While two (LOC_Os07g02800 and LOC_Os05g10670) of these TFs were expressed in *A. brasilense*-treated salt-stressed rice roots, the WRKY108 TF (LOC_Os01g60600) was not expressed in these samples. There were a few other TFs (e.g., LOC_Os12g39640, LOC_Os01g09280, LOC_Os06g30860, LOC_Os05g46020) which were differentially expressed in *A. brasilense*-treated salt-stressed rice roots. The expression pattern of these TFs suggests they likely play crucial roles in regulating the plant’s responses to salt and *A. brasilense*.

### 4.2 Expression of genes in rice-*Azospirillum brasilense* interaction

Previously, we identified several defense-related genes, flavonoid biosynthesis pathway genes, receptor-like kinases, and nitrate and sugar transporters differentially expressed in rice roots upon inoculation with *A. brasilense* at one- and fourteen- dpt (Thomas et al., 2019). Therefore, it is no surprise that in the current study, some of these genes were also differentially expressed in *A. brasilense*-treated rice roots at seven dpt. Here we compared the expression pattern of some of these genes across the three different treatments (Supplementary Table 6).

#### 4.2.1 Defense- and stress– related genes

Defense- and stress-related genes are usually differentially expressed in plants upon both abiotic (e.g., salt, heat) and biotic (e.g., bacteria, fungi) exposure (Atkinson and Urwin, 2012). For instance, defense- and stress-related genes are differentially expressed in plants when exposed to salt stress (Kumar et al., 2013; Zhang et al., 2017; Ma et al., 2022). Interestingly, multiple studies have reported that host plants suppress the expression of some of their defense-related genes during beneficial plant-microbe interactions (Soto et al., 2009; Toth and Stacey, 2015; Thomas et al., 2019; Mukherjee, 2022; Wiggins et al., 2022). For instance, during rice-*A. brasilense* interactions, defense genes such as thionins, pathogenesis-related (PR) genes, chitinases, and cinnamoyl-CoA-reductase genes were differentially expressed in rice roots (Thomas et al., 2019). In this study, we identified the expression pattern of some defense genes under the three treatments. For instance, we observed a chitinase gene (LOC_Os05g33140) upregulated in expression under all three treatments, suggesting it is not specific for either *A. brasilense* or salt treatment. Interestingly, this gene’s expression was much higher in *A. brasilense*-treated salt-stressed plants than in the other plants. We found a thionin gene (LOC_Os06g31280) upregulated in expression to a similar level in both salt treatments (salt only and salt + *A. brasilense*) but not differentially expressed in rice roots inoculated with *A. brasilense* only, suggesting its expression is specific for abiotic stress response. Next, we identified three defense-related genes [a stress-induced protein (LOC_Os01g53790), a pathogenesis-related (PR) gene (LOC_Os04g50700), and a cinnamoyl-CoA-reductase gene (LOC_Os08g34280)] downregulated in expression in *A. brasilense*-treated rice but not differentially expressed in either salt treatment (salt only and salt + *A. brasilense*). These genes were also downregulated in expression in *A. brasilense*-treated rice at fourteen dpt (Thomas et al., 2019). The expression pattern of these genes under these treatments highlights the specificity of these genes for rice-*A. brasilense* association. In conclusion, we observed differential expression of some defense- and stress-related genes under the three treatments. While some genes displayed an expression pattern specific to the salt treatment, others displayed an expression pattern specific to the *A. brasilense* treatment.

#### 4.2.2 Genes involved in the flavonoid biosynthesis pathway and nutrient transport (nitrate, ammonium, and sugar)

Past reports have suggested the possible involvement of flavonoids during the rice- *A. brasilense* interaction (Thomas et al., 2019; Mukherjee, 2022; Wiggins et al., 2022). In this study, we identified several genes from the flavonoid biosynthesis pathway differentially expressed in rice roots upon *A. brasilense* treatment. For example, a chalcone and stilbene synthase gene (LOC_Os07g34260), a key enzyme in the flavonoid biosynthesis pathway, was upregulated in expression in rice roots upon *A. brasilense* treatment. However, this gene was not differentially expressed under the two salt treatments (salt only and salt + *A. brasilense*), suggesting that the high salt conditions likely interfered with its expression pattern. We observed a similar expression pattern with other genes (e.g., LOC_Os05g41645, LOC_Os10g39140) from the flavonoid pathway. In plant-PGPB interactions, the growth promotion effects the host plants experience are due to multiple factors, including improved nutrient uptake facilitated by transporters. Previous transcriptomic studies identified the expression of nitrate, ammonium, and sugar transporters in rice during interactions with *A. brasilense* (Thomas et al., 2019; Wiggins et al., 2022). In this study, we identified two nitrate transporters (LOC_Os02g38230 and LOC_Os01g50820) upregulated in rice roots treated with *A. brasilense* only. These two nitrate transporters were also upregulated in rice roots treated with *A. brasilense* at one- and fourteen- dpt suggesting their significance in this interaction (Thomas et al., 2019). In *A. brasilense*-treated salt-stressed rice the gene encoding the nitrate transporter (LOC_Os02g38230) was upregulated in expression, but not differentially expressed in plants exposed to salt stress only. In addition, we identified an ammonium transporter (LOC_Os12g08130) and a sugar transporter (LOC_Os11g05390) upregulated in expression in rice roots treated with *A. brasilense*. Interestingly, both transporters were also upregulated in expression in the *A. brasilense*-treated salt-stressed rice plants, while showing no differential expression in rice roots treated only with salt. Overall, these findings indicate that, although some *A. brasilense*-associated genes were regulated upon high salt treatment, key transporter genes likely involved in the rice-*A. brasilense* association were also expressed under salt stress. Furthermore, the expression profile of these nutrient transporters reinforces the phenotypic observations made earlier, where inoculation with *A. brasilense* improved plant growth in rice.

### 4.3 Genes involved in hormone signaling

Plant hormones, such as auxin and ethylene, are necessary for numerous biological processes, including growth, development, signaling, and response to stress (Ferguson and Mathesius, 2014; Verma et al., 2016; Waadt et al., 2022). In fact, different plant hormones mediate salt stress responses to regulate plant growth adaptation (Yu et al., 2020). Similarly, a few reports have also elucidated the importance of hormone signaling during plant-PGPB interactions (Camilios-Neto et al., 2014; Brusamarello-Santos et al., 2019; Thomas et al., 2019; Mukherjee, 2022; Wiggins et al., 2022). Therefore, genes involved in different hormonal pathways will likely be regulated when plants are exposed to salt stress and *A. brasilense*. Here we compared the expression pattern of some genes from hormone signaling pathways across the different treatments (Supplementary Table 6).

Auxins are major regulators of plant growth and development and responses to diverse biotic and abiotic stresses (Verma et al., 2016; Waadt et al., 2022). Naturally, they are involved in plant development in response to salt stress conditions. Some studies have indicated that under salt stress, plants have decreased auxin levels and auxin transporter expression (Du et al., 2012; Liu et al., 2015). In this study in plants under salt stress-only conditions, we observed a diverse array of auxin-related genes (e.g., auxin response factors, auxin-induced proteins, auxin efflux carriers, Auxin-responsive *SAUR* genes) differentially expressed. A good portion of these genes was downregulated in expression. Auxin is also involved in different plant-PGPB interactions. In some plant-PGPB interaction reports, some auxin-responsive genes were downregulated in expression (Thomas et al., 2019; Mukherjee, 2022; Wiggins et al., 2022). We observed some genes involved in auxin signaling and biosynthesis (e.g., auxin-responsive protein, auxin-induced protein, flavin monooxygenase) downregulated in expression in *A. brasilense*-treated plants. Surprisingly, there was almost no overlap in the expression of these auxin-related genes between salt-only and *A. brasilense*-only treatments. Nearly all the genes (e.g., LOC_Os04g36054, LOC_Os01g36560, LOC_Os01g70050, LOC_Os12g43110) differentially expressed in salt stress-only treatment were not differentially expressed in response to *A. brasilense*-only treatment, and *vice versa* (e.g., LOC_Os08g44750, LOC_Os04g03980, LOC_Os01g16714). This expression pattern suggests that auxin signaling pathways mediating the plant responses and adaptation to salt and *A. brasilense* are likely separate. Furthermore, we noticed a consistent pattern in the expression of these genes (e.g., LOC_Os09g32770, LOC_Os08g42198, LOC_Os06g07040, LOC_Os01g55940) in plants exposed to both salt and *A. brasilense*. Almost all the genes differentially expressed under the combination treatment were expressed in the salt- or *A. brasilense*-only treatment and displayed the same expression pattern. These observations suggest that auxin-related genes specific to salt and *A. brasilense* treatments affect the rice transcriptome and mediate the plant’s responses and adaptations.

Ethylene is another major plant hormone that regulates multiple aspects of plant biology, including responses to biotic and abiotic stresses (Verma et al., 2016; Chen et al., 2022; Waadt et al., 2022). These studies have reported that ethylene levels are increased in plants leading to impaired growth in response to different stresses. The 1-aminocyclopropane-1-carboxylate (ACC) oxidase gene is essential for the ethylene biosynthesis pathway (Houben and Van De Poel, 2019). In this study, we observed several genes encoding ACC (e.g., LOC_Os09g27750, LOC_Os04g10350, LOC_Os08g30210, LOC_Os05g05680) differentially expressed in rice exposed to salt stress only but not expressed in *A. brasilense*-treated rice plants. Similarly, we observed differential expression of two ACC genes (LOC_Os02g53180 and LOC_Os11g08380) in *A. brasilense*-treated plants, but not in plants exposed to salt stress only. A similar expression pattern was also observed for other ethylene-related genes (e.g., LOC_Os04g41570, LOC_Os07g06130, LOC_Os04g08740). These findings are similar to our earlier observation with auxin-related genes and suggest there are separate ethylene signaling pathways mediating the plant’s responses to salt and *A. brasilense*. Furthermore, in plants exposed to salt and *A. brasilense*, both classes (salt-specific and *A. brasilense*-specific) of genes (e.g., LOC_Os04g10350, LOC_Os08g30210, LOC_Os11g08380) were differentially expressed. Overall, these findings signify that regulation of phytohormone pathways in rice roots is essential for maintaining the beneficial association with *A. brasilense* and improving plant growth under salt stress.

### 4.4 Conclusion

Our findings indicate that the plant growth-promoting bacterium *A. brasilense* improves growth in salt-stressed rice. This opens the possibility of using this PGPB to mitigate salt stress in salt-sensitive crops. Our transcriptomic data suggest that *A. brasilense* improves rice growth under salt stress by regulating the expression of key genes involved in defense and stress response, abscisic acid and jasmonic acid signaling, and ion and nutrient transport. Our results also emphasize that genes in the auxin and ethylene signaling pathways are critical for the interaction between rice and *A. brasilense* under salt stress. In this study, we collected the transcriptomic data at seven days post-treatment. Future studies can identify gene expression changes at other time points. One limitation of the study is that it was performed under *in-vitro* conditions, which do not represent actual field conditions. Similar gene expression studies can be performed under field conditions in the future. Nevertheless, our findings provide important insights into salt stress tolerance and adaptation in rice by *A. brasilense*. Using alternative approaches like PGPB will play an important role in growing crops sustainably under stressful environmental conditions.

## Supplementary Material

Supp Table 1 **SUPPLEMENTARY TABLE 1** List of differentially expressed genes (DEGs) in rice roots in response to *A. brasilense*, seven days post treatment. The table contains the gene annotations, their log_2_ fold change values, and corresponding statistics.

Supp Table 3 **SUPPLEMENTARY TABLE 3** List of differentially expressed genes (DEGs) in rice roots in response to 200 mM NaCl and *A. brasilense*, seven days post treatment. The table contains the gene annotations, their log_2_ fold change values, and corresponding statistics.

Supp Table 2 **SUPPLEMENTARY TABLE 2** List of differentially expressed genes (DEGs) in rice roots in response to 200 mM NaCl salt, seven days post treatment. The table contains the gene annotations, their log_2_ fold change values, and corresponding statistics.

Supp Table 5 **SUPPLEMENTARY TABLE 5** List of primers used in the RT-PCR experiments. The sequences of forward and reverse primers for each gene used in the RT-PCR experiments are included.

Supp Table 4 **SUPPLEMENTARY TABLE 4** List of some differentially expressed genes (DEGs) in rice roots across the three treatments. The list contains the common differentially expressed genes (228) in all the treatments, the genes (689) commonly expressed in salt and salt + *A. brasilense* treatments, and the genes (98) commonly expressed in *A. brasilense* and salt + *A. brasilense* treatments.

Supp Table 6 **SUPPLEMENTARY TABLE 6** List of some differentially expressed genes (DEGs) in rice roots across the three treatments. The table contains a list of some genes involved in salt-stress response and tolerance, rice- *A. brasilense* interaction, and hormone signaling, and their corresponding log_2_ fold change values across the three treatments (*A. brasilense* only, salt only, salt and *A. brasilense*).

## Figures and Tables

**Table 1:** Sequence Data Summary

Sample	# Reads	Reads surviving QC	% Mapped read pairs
Cnt1	21,082,666	20,227,029	89.5
Cnt2	21,433,842	20,589,590	91.7
Cnt3	22,187,681	21,294,106	91.6
Asp1	20,486,502	19,527,559	92
Asp2	22,691,171	21,813,914	90.7
Asp3	19,956,813	19,170,007	90.6
Salt1	26,388,178	25,242,562	90.7
Salt2	22,358,055	21,495,635	90.6
Salt3	29,974,714	28,872,178	78
SaltAsp1	20,664,578	19,820,165	89.1
SaltAsp2	21,152,347	20,340,052	90.2
SaltAsp3	24,881,397	23,919,351	90.8

Summary of the number of raw paired-end reads, the number of reads that survived QC criteria, and the percent of reads that were successfully aligned to the rice genome. Library contained three biological replicates of control (Cnt), A. brasilense-treated (Asp), salt-treated (Salt), and salt- and A. brasilense-treated (SaltAsp) samples.

## Data Availability

The raw sequence data is publicly available from the Sequence Read Archive (SRA) under the BioProject accession ID PRJNA962515.
